# Turning Potential Flexibility Into Flexible Performance: Moderating Effect of Self-Efficacy and Use of Flexible Cognition

**DOI:** 10.3389/fpsyg.2018.00646

**Published:** 2018-05-04

**Authors:** Ru-De Liu, Jia Wang, Jon R. Star, Rui Zhen, Rong-Huan Jiang, Xin-Chen Fu

**Affiliations:** ^1^Institute of Developmental Psychology, Beijing Key Laboratory of Applied Experimental Psychology, Faculty of Psychology, Beijing Normal University, Beijing, China; ^2^Teachers’ College, Beijing Union University, Beijing, China; ^3^Graduate School of Education, Harvard University, Cambridge, MA, United States

**Keywords:** mathematical flexibility, potential flexibility, practical flexibility, self-efficacy, use of flexible cognition

## Abstract

This study examined the relationship between two types of mathematical flexibility – potential flexibility, which indicates individuals’ knowledge of multiple strategies and strategy efficiency, and practical flexibility, which refers to individuals’ flexible performances when solving math problems. Both types of flexibility were assessed in the domain of linear equation solving. Furthermore, two types of beliefs – self-efficacy and use of flexible cognition (UFC) – were investigated as potential moderators between potential and practical flexibility. 121 8th grade students from China took part in this study. Results indicate that potential flexibility positively predicted practical flexibility. Additionally, self-efficacy and UFC might moderate the relationship between these two types of flexibility, suggesting that potential flexibility may lead to different degrees of practical flexibility depending on different levels of beliefs. Implications of these findings for research on mathematical flexibility and for educational practice are discussed.

## Introduction

The ability to solve mathematical problems flexibly is a widely recognized and critical learning goal for students (Verschaffel et al., 2007; Woodward et al., 2012; Star et al., 2015). There is growing interest in insuring that mathematics instruction not only fosters the development of routine expertise but also helps students to build adaptive expertise, meaning the ability to solve mathematical tasks efficiently, creatively and flexibly with diverse and conceptually meaningful strategies (Baroody and Dowker, 2003; Hatano, 2003; Torbeyns et al., 2009). Adaptive expertise, and the ability to be a flexible problem solver, has been shown to have direct links to academic achievement more generally (Ai, 1999; Verschaffel et al., 2007; Latzman et al., 2010). Students who lack flexibility in problem solving have been found to have great difficulty in algebra (Kieran, 1992).

### Types of Flexibility: Potential vs. Practical

Flexibility is defined differently in different fields. It is conceptualized as cognitive flexibility (also called shifting or set shifting) in the executive function literature, which means changing perspectives or approaches to a problem and flexibly shifting between responses, attributes of stimuli, sets, strategies, or tasks (Lehto et al., 2003; Diamond, 2013; Müller and Kerns, 2015). In the realm of creativity, flexibility is defined as one dimension of divergent thinking that is typically operationalized as the number of different types of ideas (Torrance, 1974; Dietrich and Kanso, 2010). When flexibility is discussed in the context of solving mathematical problems, it is generally conceptualized as a comprehensive ability resulting from both conceptual and procedural knowledge (Baroody and Dowker, 2003; Newton et al., 2010), where conceptual knowledge is the knowledge of concepts and their interrelations and procedural knowledge is the ability to execute action sequences related to problem solving (Schneider et al., 2011). More specifically, Star and colleagues define a flexible problem solver as one who has knowledge of multiple strategies as well as knowledge of strategy efficiency (e.g., Star and Seifert, 2006; Rittle-Johnson and Star, 2007; Star and Rittle-Johnson, 2008). A flexible solver can select among several known strategies in order to identify the optimal strategy for a given problem, where the optimal strategy is often (but not always) the approach requiring the fewest steps to solve the problem (Blöte et al., 2000, 2001; Star and Rittle-Johnson, 2008).

This paper considers a relatively unexplored facet of flexibility – the distinction between what we refer to as potential flexibility and practical flexibility. Current conceptions of flexibility are somewhat ambiguous as to whether flexibility refers only to knowledge (the strategies that are known, and what the solver knows about strategy efficiency) or also includes the ability to act upon this knowledge in a flexible performance. Does a flexible solver merely know a lot about efficient strategies, or is it also core to flexibility that the solver actually chooses to solve problems using these efficient approaches? There is a rich literature in psychology that suggests that these two types of flexibility may be distinct – that learners may develop knowledge of strategies and strategy efficiency that may or may not be brought to bear in the solving of actual mathematics problems.

More specifically, psychologists have long made a distinction between competence and performance, especially in the domain of mathematics (e.g., Flavell and Wohlwill, 1969; Le Corre et al., 2006; Lobina, 2011). A general finding of this literature is that learners often have greater knowledge (competence) than they are able to display during problem solving (performance). For example, some studies have suggested that children’s failure in counting tasks was due to performance limitations and not because of a lack of understanding of counting principles (Greeno et al., 1984; Cordes and Gelman, 2005). Similarly, Heine et al. (2010) found that children gained knowledge about numerical magnitudes before they were able to make use of this knowledge in performance situations.

Extending the competence/performance distinction into the realm of flexibility, competence was defined as knowledge and the underlying capacity for its use (Le Corre et al., 2006; Lobina, 2011). Thus flexible competence should mean that learners have sufficient knowledge of multiple strategies and know which one is the best (e.g., knowledge). Performance was defined as the actual implemented operations (Lobina, 2011), meaning that flexible performance indicates that learners use the optimal strategy in actual mathematical problem solving (e.g., action). Since the competence/performance distinction has been used in prior research in mathematics (e.g., Le Corre et al., 2006), it is reasonable to consider the competence/performance distinction in the realm of mathematical flexibility. To better understand the competence/performance distinction in flexibility, here we define *potential flexibility* as knowledge of multiple strategies and strategy efficiency that corresponds to competence (knowledge of strategies), with *practical flexibility* defined as solving a given problem using the most appropriate strategy that corresponds to performance (use of strategies). Drawing upon the above psychological literature, we hypothesize that potential flexibility emerges before, and contributes to, practical flexibility. Note that research on mathematical flexibility has explored both practical and potential flexibility (e.g., Star and Rittle-Johnson, 2008) but has neither examined the relationship between the two nor sought explanations for why individuals might have different degrees of potential and practical flexibility. For example, Star and Rittle-Johnson (2008) reported that students who were prompted during a problem-solving intervention to solve equations using multiple strategies and also received a brief period of direct instruction on efficient strategies scored an average of 57% correct on a potential flexibility measure, yet these same students only used more efficient strategies (e.g., practical flexibility) on 22% of post-test problems.

### Factors Impacting the Potential/Practical Flexibility Distinction

Prior work in other problem-solving domains provides some explanations for why potential flexibility might emerge before, and contribute to, practical flexibility. For example, there is evidence that the strategy chosen by a learner on a given problem is impacted by the most recently used strategy (Schillemans et al., 2009; Lemaire and Lecacheur, 2010). Problem solvers may prefer to repeatedly use the same strategy across many items, even when they are aware of and can use alternative and more efficient strategies (e. g., Luwel et al., 2003; Schillemans et al., 2011; Lemaire and Leclère, 2014). Similarly, switching between strategies (e.g., between a regularly used strategy and an infrequently used alternative that may be more efficient for a given problem) entails a cognitive cost that may result in longer response times on solving problems (Luwel et al., 2009; Lemaire and Lecacheur, 2010; Ardiale et al., 2012; Taillan et al., 2015). Thus it may not be surprising that individuals persist in using one general but not always optimal strategy for solving a group of mathematics problems, even when they have knowledge of more efficient alternatives; in such cases, practical flexibility would appear to be low, despite high potential flexibility.

Strategy repetition and strategy switch costs may help to understand why potential flexibility is distinct from practical flexibility. However, another explanation points to the potential role of contextual variables in the development of flexibility (Verschaffel et al., 2009), suggesting that individuals’ beliefs have an impact on strategy choices and thus on potential and practical flexibility. Specifically, the literature indicates that two types of beliefs may serve as potential moderators in explaining the difference between individual’s potential and practical flexibility. The first one is self-efficacy – belief in one’s ability to perform flexibly; and the second is one’s habit to utilize strategy knowledge on tasks.

With respect to the former, self-efficacy has been identified as one of the most powerful beliefs related to mathematics performance and problem solving (Stevens et al., 2004; Schommer-Aikins et al., 2005; Hoffman and Spatariu, 2008). According to social cognitive theory, self-efficacy is conceptualized as the belief in one’s capabilities to successfully conduct specific tasks (Bandura, 1977a). Ample evidence demonstrates that self-efficacy positively predicts students’ mathematics performances (e. g., Stevens et al., 2004; Galla et al., 2014; Lee et al., 2014). In particular, Hoffman and Spatariu (2008) investigated the influence of self-efficacy on mental multiplication problems. In their study, accuracy, response time, and problem-solving efficiency (number of correct responses divided by response time) were all measured, and the results showed that self-efficacy increased both math problem-solving performance and efficiency. Hoffman and Spatariu (2008) suggested that the facilitating effect of self-efficacy on mathematical problem-solving efficiency came as a result of motivating the problem solver in positive ways, such as using cognitive and self-regulatory skills.

Since self-efficacy has been shown to impact mathematical problem-solving performance and efficiency, it is reasonable to investigate its effect on mathematical flexibility. Social learning theory (Bandura, 1977b) posits that whether a learner puts what s/he has learned into action is regulated to a large extent by her/his anticipation of the consequence of her/his actions. With respect to flexibility, the theory might suggest that students who believe they are flexible expect to demonstrate flexibility in their performances and thus may devote more effort to problem solving, may be more likely to experiment with more strategies, and may more confidently try unusual but efficient strategies. In other words, self-efficacy may be a potential moderator of the relationship between potential and practical flexibility, in that high potential flexibility may be more strongly linked to high practical flexibility, for individuals with high self-efficacy.

Related to but distinct from self-efficacy, another candidate belief that may explain the relationship between potential and practical flexibility comes from research on creativity and is known as habit or awareness to use one’s knowledge on tasks. Researchers who study creativity have identified a belief known as Use of Creative Cognition (UCC), where UCC indicates individuals’ ability or willingness to deploy their creativity on specific tasks (Rogaten and Moneta, 2015a,b). Somewhat akin to the competence/performance distinction described above, individuals who are high in creativity do not always perform creatively in work or study contexts (de Acedo Lizarraga and de Acedo Baquedano, 2013; Rogaten and Moneta, 2015b). Thus, it may be that the UCC in a particular context – rather than creativity more generally – influences individuals’ creative performances in that context. UCC may moderate the relationship between an individual’s creativity and whether this creativity is evident in a particular performance.

Arguably, mathematical flexibility and creativity are similar, in that both involve a series of similar cognitive processes. For creativity, idea generation and idea evaluation are two core cognitive processes (Reiter-Palmon and Illies, 2004). Specifically, there are two steps for creative problem solving: the problem solver needs to first generate multiple ideas or strategies, and then he/she needs to evaluate the ideas generated and select the best one (Herman and Reiter-Palmon, 2011). Similarly, the two key features of flexibility are generating multiple strategies (similar with idea evaluation in creativity) and choosing the optimal one through evaluating strategy efficiency (similar with idea evaluation in creativity), respectively (Star and Rittle-Johnson, 2008). Therefore, given the evidence supporting the moderating effect of UCC on creative performances, it seems plausible to consider whether a variant of UCC specifically geared to flexibility – which we refer to *Use of Flexible Cognition* (UFC), defined as the habit to deploy potential flexibility on certain mathematics problems – has a similar impact on practical flexibility. We hypothesize that for individuals with high UFC, their potential flexibility would be a significant predictor of their practical flexibility. Thus, UFC may be a potential moderator between potential flexibility and practical flexibility.

### The Present Study

This study investigated mathematical flexibility, particularly the relationship between potential and practical flexibility, in the domain of linear equation solving. We choose equation solving because it is a foundational mathematical skill for middle school students; in addition, this domain has also been used successfully in several prior studies of mathematical flexibility (e.g., Star and Seifert, 2006; Rittle-Johnson and Star, 2007; Star and Rittle-Johnson, 2008; Rittle-Johnson et al., 2012). There are a variety of solution methods for solving linear equations, with some strategies more efficient than others for some problems – which makes this domain useful for investigating flexibility.

The current study aimed at examining the moderating effects of self-efficacy and UFC between potential flexibility and practical flexibility. Based on the existing literature, it was hypothesized that potential flexibility, self-efficacy, and UFC would positively predict practical flexibility (H1); self-efficacy would moderate the relationship between potential flexibility and practical flexibility (H2); and similarly, UFC would also play a moderating role between potential flexibility and practical flexibility (H3).

## Materials and Methods

### Participants

A total of 121 Chinese students in grade eight volunteered to participate in this study. They were from a medium-sized city in East China. 54% of the participants were female (*n* = 65). The students were on average 13.72 years of age (*SD* = 0.35) with a range from 13 to 15 years old. At the time of the study, all of the participants had already learned basic knowledge about solving linear equation in one unknown, as this topic is part of the standard curriculum in the 6 and 7th grade in China.

### Ethics Statement

This study was approved by the Research Ethics Committee of Beijing Normal University and the principals of the participating schools. Written informed consent was obtained from all individual participants and their parents. All participants were informed that they had the right to withdraw from this study at any time.

### Procedure

Participants completed all assessments individually, during their regular mathematics class, under the supervision of trained research assistants. Students were first asked to complete a beliefs assessment that included scales for self-efficacy and UFC (described below). Students were given 15 min to complete the beliefs assessment; all students finished in the allotted time. Then, the mathematical flexibility assessment (which was designed to assess both potential and practical flexibility, described below) was administered. Participants were given 45 min to work on the assessment; all students finished in the allotted time. After completing the mathematical flexibility assessment, participants were thanked. Finally, the research assistants collected each student’s score on the most recent exam from their mathematics teachers, to serve as a covariate for mathematics achievement.

### Measures and Coding

#### Self-Efficacy

Four items were used to assess participants’ self-efficacy on their flexibility. These items were based on social cognition theory (Bandura, 1977a) but with modifications in wording to specifically target mathematical flexibility. The four items were (a) “I can think of multiple methods when solving a math problem,” (b) “I can tell which method is the best when solving a math problem,” (c) “I believe that I am flexible when solving math problems,” and (d) “I can think of multiple ways to solve a math problem, and choose the best one.” Responses were rated on a Likert-scale of 1 (not true at all) to 7 (very true). Each student’s self-efficacy score was calculated as the average of the four items. The internal reliability of the scale in this study was good (α = 0.84), and the fit indices of the confirmatory factor analysis were acceptable, χ^2^/*df* = 1.812, CFI = 0.996, TLI = 0.974, RMSEA = 0.082.

#### Use of Flexible Cognition (UFC)

Participants reported their UFC for solving equations using six items. These items were based on the work of Rogaten and Moneta (2015a), with some modifications to specifically target mathematical flexibility as well. The six items were (a) “I notice the algebraic structures on both sides of the equation when solving equations,” (b) “I notice the operational relations among the numbers in the equation when solving equations,” (c) “The method I use to solve the problem is chosen from several methods that I had thought of,” (d) “I tend to use the simplest method when solving equations,” (e) “I do not observe the equation. I just followed the procedure step by step to solve equations (R),” and (f) “I do not bother to think of simpler ways to solve equations (R).” Responses were recorded on a Likert-scale of 1 (not true at all) to 7 (very true). The UFC score for each student was calculated as the average of the six items. The internal reliability of the scale in this study was acceptable (α = 0.61), and the fit indices of the confirmatory factor analysis were good, χ^2^/*df* = 1.154, CFI = 0.993, TLI = 0.986, RMSEA = 0.036.

#### Mathematical Flexibility Assessment

The mathematical flexibility assessment (Xu et al., 2017) consisted of 12 linear equations (see **Table 1**). Drawing on prior research on flexibility in linear equation solving (e.g., Star and Seifert, 2006; Rittle-Johnson and Star, 2007), the assessment included four types of equations, each of which can be solved using both a standard algorithm and a more innovative approach that takes advantage of specific numerical or structural features of the problem to arrive at a solution more efficiently.

**Table 1:** Equations with standard algorithms and innovative methods.

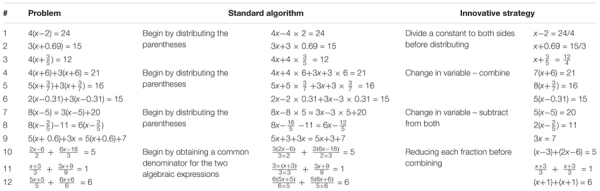

The first three problem types were taken directly from Star and Seifert (2006) and included problems such as 4(*x* – 2) = 24, 4(*x* + 6) + 3(*x* + 6) = 21, and 8(*x* – 5) = 3(*x* – 5) + 20. The standard algorithm for solving these types of linear equations (see **Table 1**) involves first distributing the parentheses, then combining like terms on either side of the equals sign, then collecting variable terms to one side and constant terms to the other side of the equals sign. Yet for each of these types, there exists a more innovative strategy. This innovative strategy for the equation 4(*x* – 2) = 24 involves dividing both sides by 4 as a first step before distributing. For the equations 4(*x* + 6) + 3(*x* + 6) = 21 and 8(*x* – 5) = 3(*x* – 5) + 20, the innovative strategy involves first combining like variable terms [(*x* + 6) and (*x* – 5) respectively] before distributing. The fourth type of problem was inspired by Newton (2008) and involved solving equations that included fractions such as (2*x* – 6)/2 + (6*x* + 18)/3 = 5. A standard algorithm for solving these types of equations would begin with obtaining a common denominator to add the fractional expressions first, while a more innovative strategy would involve reducing each fraction first, which serves to eliminate the fractional expressions and enables the equation to be solved more simply. Note that on all four problem types, it is usually possible to determine whether a solver has used a standard algorithm or an innovative strategy by analyzing the first one or two steps of their solution method.

Students made three passes through these 12 problems, in order to provide data for assessing both potential and practical flexibility. First, students were asked to solve each problem quickly and accurately. After completing all 12 problems, students were asked to return to the beginning of the test and, for each problem, to generate as many additional, different strategies for each problem as possible. Finally, students were asked to return again to the beginning of the test and to select (from among the multiple strategies that they had produced for each problem) the one strategy that they felt was optimal for that problem.

Students’ solution methods were coded by trained research assistants who were doctoral students in mathematics education. For each problem, coders determined each of the following. First, coders determined whether each student used standard and/or innovative strategies on each problem, by looking at the multiple strategies generated for each problem in both the first and the second pass through the assessment. For each student, each problem was coded as indicating knowledge of the standard algorithm, the innovative strategy, both, or neither. Second, coders looked specifically at the solution method generated in the first pass through the assessment and judged whether each student’s first solution to each problem used the standard algorithm, an innovative strategy, or neither. Finally, for each problem coders looked at the strategy that each student selected in the third pass through the assessment (where students were asked to identify an optimal strategy from among the methods that they had generated) and determined whether the student-identified optimal strategy aligned with the standard algorithm, the innovative strategy, or neither. As has been done in prior work on flexibility in linear equation solving (e.g., Star and Seifert, 2006; Rittle-Johnson and Star, 2007), whether or not students arrived at the correct numerical solution for an equation was not used in the coding of strategies.

#### Potential Flexibility

Potential flexibility is defined here as students’ knowledge of multiple strategies and knowledge of strategy efficiency. For each problem, if a student demonstrated knowledge of both the standard algorithm and the innovative strategy, and if the student was able to select (in the third pass through the assessment) the innovative strategy as optimal, the student was deemed to have potential flexibility for that problem, earning a score of 1. If a student only showed knowledge of either the standard algorithm or the innovative strategy (but not both) for a given problem, or the student did not select an innovative strategy as optimal for that problem, the student earned a Potential Flexibility score of 0 for that problem. Potential flexibility scores for the 12 assessment problems were added together, resulting in an overall Potential Flexibility score for each student that ranged from 0 to 12.

#### Practical Flexibility

Practical flexibility – whether a student had the ability to use innovative strategies in a performance – was calculated by determining whether each student used the innovative strategy for each problem on their first attempt (e.g., in the first pass) through the assessment. If coders determined that the first attempt was the innovative strategy, the student earned a Practical Flexibility score of 1 for that problem. Otherwise, the Practical Flexibility score was 0 for that problem. Practical flexibility scores for the 12 assessment problems were added together, resulting in an overall Practical Flexibility score for each student that ranged from 0 to 12.

#### Covariate

Two variables (mathematics achievement and procedural skill) were used as covariates in the analysis, to assess participants’ mathematical capabilities. For mathematics achievement, we used each student’s score on the class’s most recent mathematics exam. Procedural skill indicated whether students were able to correctly solve the 12 linear equations on the assessment. For each equation, and looking only at the solution method used by the student on the first pass through the assessment, participants were given 1 point for each correct numerical solution and 0 points otherwise. Overall Procedural Skill scores for each student ranged from 0 to 12.

### Data Analyses

Descriptive statistics and correlations between the major variables were examined. Then, a paired-samples *T*-test was conducted to compare potential flexibility and practical flexibility scores. Next, moderating effects were examined by a hierarchical regression with two models (Dawson, 2014). First, the main effects model (model 1) was conducted, wherein participants’ practical flexibility scores were regressed on the covariate and the predictors (mathematics achievement, procedural skill, self-efficacy, UFC, and potential flexibility). The interaction terms were added in model 2, which included the interactions of potential flexibility with each belief predictor (self-efficacy and UFC). The covariate and independent variables were centered according to the procedures of testing interactions (Aiken and West, 1991). Considering that moderating effects were exploratory in nature, this study used a.10 alpha level to interpret the interactions (Durand, 2013; Blankson and Blair, 2016). Significant interactions were tested by computing simple slopes at one standard deviation above and below the means of self-efficacy and UFC (Aiken and West, 1991; Dawson, 2014).

## Results

Results of descriptive statistics are shown in **Table 2**. There is no missing data. Participants showed moderate levels of practical flexibility (*M* = 6.31, *SD* = 3.99) and comparatively high levels of potential flexibility (*M* = 8.79, *SD* = 3.35). Results of the paired-samples *T*-test showed that participants earned significantly higher potential flexibility scores than practical flexibility scores (*t* = 6.21, *p* < 0.001, *Cohen’s d* = 0.56). Correlations among study variables are shown in **Table 3**. Results indicated that self-efficacy (*r* = 0.18, *p* < 0.05), UFC (*r* = 0.18, *p* < 0.05), and potential flexibility (*r* = 0.30, *p* < 0.01) were all positively correlated with practical flexibility, with the relation between potential flexibility and practical flexibility as the strongest.

**Table 2:** Descriptive statistics for major variables.

Variable	Statistic				
	*N*	Min.	Max.	Mean	*SD*
Achievement	121	68	118	103.88	11.43
Procedural skill	121	5.00	12.00	10.84	1.44
Self-efficacy	121	1.00	7.00	5.34	1.19
UFC	121	4.00	7.00	5.98	0.75
Potential flexibility	121	0.00	12.00	8.79	3.35
Practical flexibility	121	0.00	12.00	6.31	3.99

**Table 3:** Correlations among major variables.

Variable	1	2	3	4	5
(1) Achievement	1				
(2) Procedural skill	0.44^∗∗^	1			
(3) Self-efficacy	0.31^∗∗^	0.18^∗^	1		
(4) UFC	0.25^∗∗^	0.23^∗^	0.54^∗∗^	1	
(5) Potential flexibility	0.38^∗∗^	0.42^∗∗^	0.32^∗∗^	0.33^∗∗^	1
(6) Practical flexibility	0.23^∗^	0.21^∗^	0.18^∗^	0.18^∗^	0.30^∗∗^

*^∗∗^*p* < 0.01. ^∗^*p* < 0.05.*

### Self-Efficacy and UFC as Potential Moderators Between Potential and Practical Flexibility

The results for multiple regression analysis are presented in **Table 4**. As expected, the main effect of potential flexibility was significant, indicating that potential flexibility was a significant predictor of practical flexibility. The result indicated that participants who were higher in potential flexibility were indeed higher in practical flexibility. Additionally, it is worth noting that the potential flexibility × self-efficacy and potential flexibility × UFC interactions were also significant in a.10 alpha level.

**Table 4:** Regression analyses for effects on practical flexibility.

Model	(1). Main effect			(2). Interaction		
Variable	B	SE	*Sig.*	B	SE	*Sig.*
Achievement	0.41	0.40	0.31	0.57	0.38	0.14
Procedural skill	0.25	0.40	0.54	0.05	0.39	0.89
Self-efficacy	0.18	0.42	0.67	0.21	0.42	0.62
UFC	0.19	0.41	0.65	0.30	0.43	0.49
Potential flexibility	0.81	0.40	0.04^∗^	1.09	0.39	0.006^∗∗^
Potential flexibility × self-efficacy				0.66	0.35	0.06
Potential flexibility × UFC				0.63	0.37	0.09

	**Variance component**			**Variance component**		

Residual	13.97			12.79		
Intercept	6.31			5.90		

	**Model fit**			**Model fit**		

Log likelihood	-331.21			-325.90		
R^2^	0.11			0.19		

*^∗∗^*p* < 0.01. ^∗^*p* < 0.05.*

**FIGURE 1:**
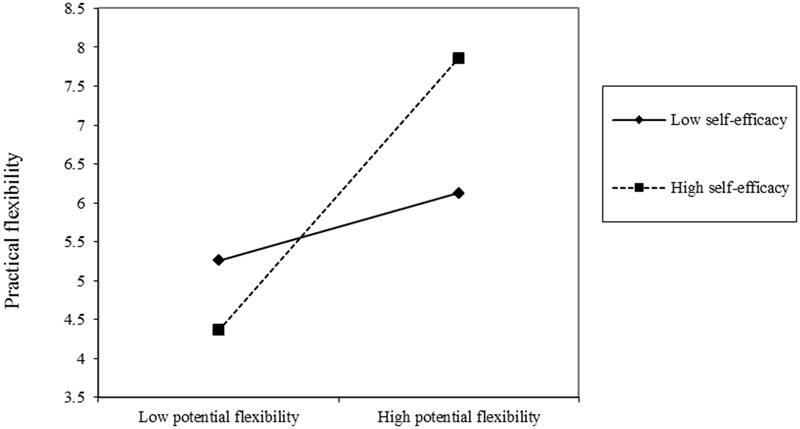
Practical flexibility as a function of potential flexibility and self-efficacy.

**FIGURE 2:**
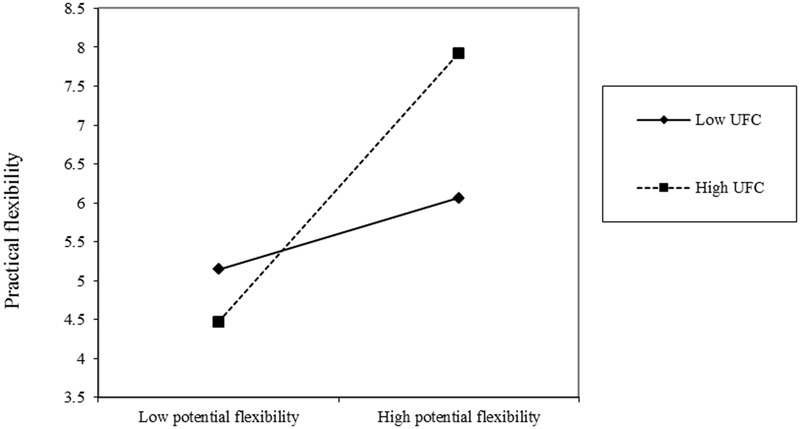
Practical flexibility as a function of potential flexibility and UFC.

Simple slopes at one standard deviation above and below the means of self-efficacy and UFC were calculated to further examine the two-way interactions (Aiken and West, 1991; Dawson, 2014). Participants higher in potential flexibility showed more practical flexibility with high self-efficacy (*simple slope t* = 3.02, *p* = 0.003; see **Figure 1**). However, potential flexibility did not significantly influence practical flexibility of participants with low self-efficacy (*simple slope t* = 0.85, ns; see **Figure 1**). Similarly, in the high UFC group, participants with higher levels of potential flexibility performed more flexibly compared to participants with lower potential flexibility (*simple slope t* = 2.93, *p* = 0.004; see **Figure 2**), whereas potential flexibility was not a significant predictor of practical flexibility in low UFC group (*simple slope t* = 0.86, ns; see **Figure 2**). These results were consistent with our hypothesis regarding the moderating effect of self-efficacy and UFC on the relation between potential flexibility and practical flexibility.

## Discussion

Flexibility is considered an important aspect of mathematical competence, playing a crucial role in all aspects of mathematics (Heinze et al., 2009). A proficient solver should have knowledge of multiple strategies as well as the ability to evaluate the strength and weakness of each strategy according to the particular characteristics of different problems. However, a common concern is that solvers do not use the most efficient strategy as their first choice when solving specific mathematic problems, even if they appear to have knowledge of more efficient strategies (Lemaire and Siegler, 1995; Luwel et al., 2003, 2009; Rieskamp and Otto, 2006; Lemaire and Lecacheur, 2010). Why is it so hard for students to put their knowledge into action – to demonstrate not only potential flexibility but also practical flexibility? A key contribution of this study was the identification of these two types of mathematical flexibility: Practical flexibility reflects an individual’s flexible performance when solving certain mathematics problems, whereas potential flexibility indicates an individual’s competence or knowledge of strategies and strategy efficiency.

An additional contribution of this study was the development of an assessment protocol for measuring potential and practical flexibility. Students were given a series of equations to solve and asked to make several passes through the equations – first solving each one, then generating as many alternative strategies as possible for each equation, and finally selecting the most optimal strategy from among generated strategies. Participants’ practical flexibility scores were assessed by whether they used the most efficient method on their first attempt at solving an equation, which focused on their flexible performance (e.g., using the best strategy in actual equation solving practice); potential flexibility was assessed by whether students could generate multiple solutions and identify innovative strategies from among the methods that they had generated, which focused on their flexible competence (e.g., having knowledge of multiple strategies and strategy efficiency). This method of assessing flexibility allowed for an examination of the relationship between potential and practical flexibility. Results from a paired-samples *T*-test showed that participants’ potential flexibility scores were significantly higher than their practical flexibility scores, which showed that individuals with high levels of flexible competence might display lower levels of flexible performance. These results are consistent with prior psychological research on the competence/performance distinction. Furthermore, we found that individuals with higher levels of potential flexibility showed more practical flexibility compared with those who had lower potential flexibility.

The present study also investigated two aspects of students’ beliefs (self-efficacy and UFC) as potential moderators of the relationship between potential and practical flexibility. Results showed that the two-way interactions of potential flexibility × self-efficacy and potential flexibility × UFC were both significant. The effect of potential flexibility on practical flexibility was seen among students with higher levels of self-efficacy. Students with higher potential flexibility may be hindered in their practical flexibility performance if they do not believe they can generate multiple strategies, or have no confidence to their own judgment about strategy efficiency. Students with lower levels of self-efficacy may choose not to try different strategies, perhaps because they are concerned about their perceived lack of ability, thus leading to poorer practical flexibility. Similarly, UFC was also found to be a potential moderator between potential flexibility and practical flexibility. Specifically, individuals with high potential flexibility showed more practical flexibility when they had higher UFC for solving equations.

While this study represents an important step in deepening our understanding of mathematical flexibility, it is important to note limitations of the present work that suggest avenues for future work. First, these results should be replicated in other mathematical domains. Flexibility has great relevance for mathematics at all levels. Although this study builds on a body of work exploring flexibility in linear equation solving (e.g., Star and Seifert, 2006; Rittle-Johnson and Star, 2007; Star and Rittle-Johnson, 2008), future studies should utilize additional mathematical domains where flexibility has been investigated, including but not limited to recursion (e.g., Luwel et al., 2009; Schillemans et al., 2011), estimation (e.g., Star and Rittle-Johnson, 2009; Peters et al., 2013; Lemaire and Leclère, 2014), and differentiation (Maciejewski and Star, 2016). Second and similarly, future study should investigate flexibility in other student populations. It is possible that unique features of the educational system, culture, and commonly used pedagogies in China may have influenced these results, both in terms of students’ mathematical knowledge as well as students’ beliefs. Specifically, Imbo and LeFevre (2009) found that Chinese participants used less efficient strategies than did Belgians and Canadians participants when solving math problems. The authors argued that these results might due to the different instructiona l approaches: practice and training were favored in China, while exploration and flexibility were favored in European and North American (Imbo and LeFevre, 2009). Third, the finding about the potential moderating effect of self-efficacy and UFC is tentative as a result of the cross-sectional design of the present study. In particular, cross-sectional data is not sufficient for assuming moderating effects in a very strict sense (Kraemer et al., 2001, 2002, 2008). Kraemer et al. (2001; 2002; 2008) argued that one of the eligibility criteria for establishing a moderator was temporal precedence (the moderator precedes the independent variable in time). The design of this study makes it difficult to establish temporal precedence. A further challenge to the claim that self-efficacy and UFC are potential moderators is the finding that potential flexibility was correlated with self-efficacy and UFC. According to Kraemer et al. (2001, 2002, 2008), these associations might lead to other explanations of their relationships (e. g., that self-efficacy and UFC may have mediated the relation between potential flexibility and practical flexibility). Thus, to further prove these moderating effects, experimental longitudinal studies are needed in future work. Fourth, self-efficacy was assessed using a self-report measure in this study. Given that self-efficacy could influence individual’s performance and could also be affected by one’s performance (Bandura, 1977a), this study cannot rule out the possibility that the measured self-efficacy might be influenced by individual’s flexibility performance. As a result, in order to more strictly prove the effect of self-efficacy on practical flexibility, manipulating self-efficacy is still necessary in future studies.

## Conclusion

The findings of the present study have important implications for research on mathematical flexibility. Foremost, these results expand our understanding of mathematical flexibility by distinguishing between potential and practical flexibility. Potential flexibility, which concerns individuals’ ability to generate multiple strategies and choose the most suitable one, does not always lead to practical flexibility. Furthermore, the present study focused on factors related to the relationship between potential and practical flexibility, including the role of students’ beliefs in understanding why practical flexibility is generally lower than potential flexibility. By examining how beliefs may influence whether potential flexibility is related to practical flexibility, this study begins to unpack the factors that influence flexibility performance, which may lead to development of interventions and programs to promote students’ optimal performance in mathematics.

There are practical implications to this study as well. How to improve students’ flexibility performance in mathematics is a central issue in mathematics education (Heinze et al., 2009). Based on results of the current study, flexibility requires not only the development of sufficient knowledge and strategies, but it is also the case that changing students’ beliefs may play a strong role in helping students show more practical flexibility. Further attention should be paid to strengthening students’ confidence in their own abilities and in developing productive beliefs related to the deployment of knowledge and skills to performance on mathematics problems. The findings of the current study provide a new perspective for educators who seek to develop flexible performances in mathematics.

## Author Contributions

JW designed the study and wrote the manuscript. R-DL assisted in the design and implementation of the study, and revised the manuscript. JS assisted in the design and implementation of the study and helped in the writing and editing of the manuscript. RZ, R-HJ, and X-CF assisted in data collection and trained research assistants.

## Conflict of Interest Statement

The authors declare that the research was conducted in the absence of any commercial or financial relationships that could be construed as a potential conflict of interest.
